# On a New Formula for Giving Digitalis

**Published:** 1823-12

**Authors:** 


Art. VIII.—*On a new Formula for giving Digitalis.* By a Doctor in Medicine.

Great difference of opinion exists amongst medical men relative to the virtues of foxglove, as a remedy for the cure of diseases: some think it a very efficacious medicine; some a very uncertain one; whilst others, again, consider it as not at all deserving of the high reputation it has acquired.

Having frequently witnessed the beneficial effects of digitalis in dropsies and other diseases, particularly when the plant was exhibited in substance, and considering that its occasional failure depends more upon the formula and mode of using it, than upon any actual inefficacy of the medicine itself, the author of this communication has, with a view of obviating some of the objections that might be made against the present preparations of digitalis, been lately induced to try its action on the human frame in the form of an ethereal tincture, made in the following manner:—℞. Foliorum Digitalis, ʒj.; Spiritus Ætheris Nitrici, f.℥jss. M. et macera per dies quatuordecim, dein cola.

The principal reasons which have led to the employment of foxglove in this form of tincture, arise from the opinion for some time entertained by the author, and in which it is thought that both reason and experience will bear him out,—viz. that the common tincture of digitalis, made with proof spirit, is an incompatible and, it might be said, an unphilosophical preparation; for every tyro in medicine must know that digitalis is a debilitating remedy, and that its chief action, along with its diuretic effect, is to diminish the frequency of the pulse and stimulus of the system; whilst, on the other hand, the spirit of wine employed is a strong diffusible stimulant, and acts remarkably in accelerating the circulation of the blood, increasing the heat of the body, &c. Thus, the action of the one must consequently counteract and destroy that of the other; and, if an inert preparation is not thereby produced, it must, at all events, be allowed that a less effectual medicine is used than if digitalis was given in substance, or infused in water.

In stating this opinion, it is at the same time not overlooked that many think so small a quantity of spirits of wine can have but little, if any, effect in diminishing the efficacy of digitalis. This might be very just reasoning if the alcohol was taken in its unmixed state, or if the body was not under the influence of disease; but when it is combined with foxglove, even in a very small quantity, or when the individual is affected with disease, it may act so as materially to affect its peculiar influence on the human body: this certainly ought not to be lost sight of. That the addition of another substance, even in a small proportion, may very essentially change the active virtues of a medicine, is very fully demonstrated by the well-known fact which occurs in regard to mercury in its simple state, and when it is united with muriatic acid; for, a large quantity of the metal may be given in its pure state without producing any apparent effect on the system, but combine only a few grains of it with as many drops of muriatic acid, and we possess the most powerful mercurial known. By parity of reasoning, then, may not the same phenomena take place, when other substances are the subject of experiment?

By what has been said, it is not intended to do away with the common tincture of digitalis altogether, but only to show that it is not the most effectual, though it may be allowed to be the most convenient formula now in use for giving that medicine. The ethereal tincture is not liable to the above objections; but, whilst it possesses the convenience and other advantages of the ordinary tincture, it has also, in an eminent degree, both the diuretic and debilitating properties of that plant. Further, it at the same time combines two diuretic medicines, which, like two purgatives given together, act much more effectually and certainly than either would do if given separately.

This ethereal tincture of digitalis may have been employed by others: the author, however, is not aware that it has, nor does he recollect that it is mentioned by any writer. The Prussian Pharmacopœia does indeed give a formula made with the sulphuric, but not with the nitric, æther: however, as that tincture is liable to the same, and even to stronger objections, than the common form, (for it is still more stimulating,) it cannot be considered as a medicine of the same nature as that made with nitric æther.

A good deal of personal experience convinces the author that this mode of preparing foxglove is of considerable utility, and that it merits the attention of those medical practitioners who are anxious to augment the number and efficacy of the means which they possess for alleviating and curing the diseases of their fellow creatures.

To enumerate the complaints in which this formula should be exhibited, would only be to repeat what every one already knows: it therefore will suffice to say, that it may be given in all those cases in which digitalis is, in the present state of medical knowledge, considered to be indicated; the above preparation of that medicine being only recommended as a more efficacious, convenient, and correctly medical one, than any of those now in use.

*September* 1823.

